# A population of large neurons in laminae III and IV of the rat spinal cord that have long dorsal dendrites and lack the neurokinin 1 receptor

**DOI:** 10.1111/j.1460-9568.2007.05793.x

**Published:** 2007-09

**Authors:** Erika Polgár, Suzanne Thomson, David J Maxwell, Khulood Al-Khater, Andrew J Todd

**Affiliations:** Spinal Cord Group, Institute of Biomedical and Life Sciences, University of GlasgowGlasgow G12 8QQ, UK

**Keywords:** confocal microscopy, NF200, NK1 receptor, projection neuron

## Abstract

The dorsal horn of the rat spinal cord contains a population of large neurons with cell bodies in laminae III or IV, that express the neurokinin 1 receptor (NK1r) and have long dorsal dendrites that branch extensively within the superficial laminae. In this study, we have identified a separate population of neurons that have similar dendritic morphology, but lack the NK1r. These cells also differ from the NK1r-expressing neurons in that they have significantly fewer contacts from substance P-containing axons and are not retrogradely labelled following injection of tracer into the caudal ventrolateral medulla. We also provide evidence that these cells do not belong to the postsynaptic dorsal column pathway or the spinothalamic tract. It is therefore likely that these cells do not have supraspinal projections. They may provide a route through which information transmitted by C fibres that lack neuropeptides is conveyed to deeper laminae. The present findings demonstrate the need for caution when attempting to classify neurons solely on the basis of somatodendritic morphology.

## Introduction

Rexed (1952) divided the dorsal horn into six parallel laminae, and this scheme has been applied to other species, including rodents and humans (Schoenen, 1982; Molander *et al*., 1984). Primary afferent input to the dorsal horn is highly organized: most nociceptive afferents terminate in laminae I and II, while low-threshold cutaneous afferents occupy a region extending from the inner part of lamina II (IIi) to lamina V (Todd & Koerber, 2005). Rexed (1952) observed that lamina III contained numerous small neurons and scattered larger cells, while neurons in lamina IV were more variable in size. Early Golgi studies identified large neurons in the region corresponding to lamina IV that had dorsal dendrites extending into the superficial laminae (Ramon y Cajal, 1909; Szentágothai, 1964; Scheibel & Scheibel, 1966; Schoenen, 1982). Subsequent studies have described a population of relatively large lamina III neurons that were often pyramidal in shape, and these also had dendrites that branched extensively in laminae I and II (Réthelyi & Szentágothai, 1969; Brown, 1981).

Many nociceptive afferents contain substance P (Hokfelt *et al*., 1975; Lawson *et al*., 1997), which acts on the neurokinin 1 receptor (NK1r). Immunocytochemical studies have revealed a population of large neurons in laminae III and IV of the rat spinal cord that express the NK1r and have prominent dorsal dendrites that enter the superficial laminae (Bleazard *et al*., 1994; Brown *et al*., 1995; Littlewood *et al*., 1995; Mantyh *et al*., 1995). It has been reported that there are approximately 20 cells of this type on each side in the L4 spinal segment in the rat. These cells are known to be projection neurons, as virtually all of them can be retrogradely labelled following injection of tracer into the caudal ventrolateral medulla (CVLM), while most project to the lateral parabrachial area, and some to the periaqueductal grey matter and thalamus (Marshall *et al*., 1996; Todd *et al*., 2000).

We have recently found that the cell bodies and dendritic trees of the large lamina III/IV NK1r-expressing neurons were immunoreactive with an antibody against neurofilament 200 (NF200; Todd & Polgár, unpublished observations). However, we also observed a population of NF200-immunoreactive neurons in this region that had similar morphology, with dendrites that entered the superficial dorsal horn but lacked NK1r immunoreactivity. In order to establish whether these cells form a population that is clearly distinct from the NK1r-expressing neurons, we have examined the density of contacts that they receive from substance P-containing axons, and have investigated whether they can be retrogradely labelled from the CVLM or thalamus. We also injected tracer into the gracile nucleus to determine whether these cells belonged to the postsynaptic dorsal column (PSDC) pathway, as some PSDC neurons are located in lamina III in the rat (Giesler *et al*., 1984).

## Materials and methods

### Animals

Experiments were approved by the Ethical Review Process Applications Panel of the University of Glasgow, and were performed in accordance with the UK Animals (Scientific Procedures) Act 1986.

Twelve adult male Wistar rats (240–390 g; Harlan, Loughborough, UK) were used in this study. Three of these were deeply anaesthetized with pentobarbitone and perfused through the left ventricle with a fixative consisting of 4% freshly depolymerized formaldehyde. Six animals were anaesthetized with a mixture of ketamine and xylazine (73.3 and 7.3 mg/kg intraperitoneal, respectively, supplemented as necessary) and placed in a stereotaxic frame. Three of these rats received injections of 200 nL 1% cholera toxin B subunit (CTb, Sigma, Poole, UK) into the CVLM on the left side (Lima *et al*., 1991; Todd *et al*., 2000), while three had injections of 200 or 300 nL 1% CTb that were targeted on the left gracile nucleus. The remaining three rats were anaesthetized with halothane and received stereotaxic injections of Fluorogold (Fluorochrome, Englewood, CO, USA) into the left thalamus. In each of these cases, five injections of 100 nL of 4% Fluorogold were administered: three of these were aimed at the ventral posterior nuclei at different rostro-caudal locations; one into the medial thalamus at 5.8 mm in front of the ear-bar; and one at the triangular part of the posterior nuclear group (Gauriau & Bernard, 2004). After a 3 or 4-day survival period, the animals that had had CTb or Fluorogold injections were re-anaesthetized with pentobarbitone and perfused with fixative as described above.

### Tissue processing and immunocytochemistry

Lumbar spinal cord segments from all animals were removed and stored in the same fixative for 8–18 h, before being cut into parasagittal 60-µm-thick sections with a Vibratome. In all cases, sections were immersed in 50% ethanol for 30 min prior to immunoreaction to enhance antibody penetration (Llewellyn-Smith & Minson, 1992).

Sections from the three rats that had not received retrograde tracer injections were incubated for 72 h in a cocktail of primary antibodies that consisted of mouse monoclonal antibody directed against NF200 (Sigma, clone N52; 1 : 1000), rabbit antiserum against NK1r (Sigma; 1 : 10 000) and rat monoclonal antibody against substance P (Oxford Biotechnology, Oxford, UK, clone NC1/34 HL; 1 : 100). They were then incubated overnight in species-specific secondary antibodies raised in donkey and conjugated to Rhodamine Red, Cy5 (both from Jackson Immunoresearch, West Grove, PA, USA; 1 : 100) or Alexa 488 (Invitrogen, Paisley, UK; 1 : 500), before being mounted in anti-fade medium (Vectashield, Vector Laboratories, Peterborough, UK).

Parasagittal sections through both sides of the L4 segment from the rats that had received CTb injections into the CVLM, and through the left (ipsilateral) side of this segment from the rats that received injections into the gracile nucleus were processed for immunocytochemistry as described above, but with a primary antibody cocktail consisting of monoclonal antibody against NF200 and rabbit anti-NK1r (as above), together with goat anti-CTb (List Biological Laboratories, Campbell, CA, USA; 1 : 5000). The brainstems of the animals that had received CTb injections were cryoprotected in 30% sucrose in phosphate buffer, and in each case the region that included the injection sites was cut into 100-µm-thick coronal sections with a freezing microtome. Sections were reacted with goat anti-CTb (1 : 50 000) by using an immunoperoxidase method (Todd *et al*., 2000). All injection sites were examined, and in each case the spread of tracer from the injection site was plotted onto drawings of the brainstem (Paxinos & Watson, 1997).

Parasagittal sections through the right (contralateral) side of the L2 segment of the rats that had received injections of Fluorogold into the thalamus were processed for immunocytochemistry as described above, but with the following primary antibodies: guinea-pig anti-Fluorogold (Protos Biotech, New York, USA; 1 : 500); monoclonal anti-NF200; and rabbit anti-NK1r. The brains of these rats were cryoprotected and sectioned as described above, and the spread of Fluorogold from the injection sites was plotted onto drawings of the brain (Paxinos & Watson, 1997).

The monoclonal antibody N52 recognizes both phosphorylated and non-phosphorylated forms of neurofilaments with a molecular weight of 200 kDa. The rat substance P antibody (mAb NC1/34 HL) recognizes a sequence common to substance P and neurokinins A and B (Cuello *et al*., 1979), but appears to be relatively selective for substance P and neurokinin A when used for immunocytochemistry in these conditions (McLeod *et al*., 2000; Polgár *et al*., 2006). The NK1r antibody was raised against a peptide corresponding to amino acids 393–407 of the rat receptor, which was conjugated to keyhole limpet haemocyanin. Staining of immunoblots of rat brain membrane fractions is specifically inhibited by preabsorption of the antibody with this peptide (manufacturer's specification).

### Confocal microscopy and analysis

Cells in lamina III or IV that were immunoreactive for NF200 but not the NK1r and had prominent dorsal dendrites that entered the superficial dorsal horn were examined in sections from the three unoperated rats that had been reacted with antibodies against NF200, NK1r and substance P. The sections were first viewed with epifluorescence through a 20 × lens to allow identification of the cells, and these were then scanned through a 60 × oil-immersion lens with a Radiance 2100 confocal microscope (Bio-Rad, Hemel Hempstead, UK). Sequential scanning was used to minimize fluorescent bleed-through. Twenty-seven cells of this type were identified (between seven and 13 per rat), and z-series (0.5 µm z-separation) were obtained through the dendritic tree of each of these neurons. In each case the presence of dendrites that entered lamina II was confirmed and the depth of the soma below the white matter was measured.

To determine the density of contacts from substance P-containing axons onto the dendrites of large lamina III/IV NF200-positive cells that lacked the NK1r, we selected 15 of the 27 cells (five each from three rats) for analysis. This selection was carried out before substance P immunoreactivity was examined. Confocal image stacks (obtained as described above) were analysed with Neurolucida for Confocal software (MicroBrightField, Colchester, VT, USA). For each cell, the NF200-immunoreactive dendrites were followed through the z-series, and the location of all identified contacts on these dendrites from substance P-immunoreactive varicosities was plotted. The length of dendrites and the density of contacts per 100 µm was then determined. To allow for comparison with the results of Naim *et al*. (1997), those parts of the dendrites that lay within the plexus of substance P-immunoreactive axons (superficial dendrites) were analysed separately from parts of dendrites that lay ventral to this plexus (deep dendrites). Results for superficial and deep dendrites were compared with the corresponding values obtained for NK1r-immunoreactive lamina III/IV neurons (Naim *et al*., 1997) by using unpaired *t*-tests.

Sections from the L4 segment of the rats that had received injections of CTb into the CVLM or the gracile nucleus were examined to determine whether any of the NF200-positive/NK1r-negative cells with dendrites that entered the superficial dorsal horn were retrogradely labelled with CTb. In each case, these cells were identified (based on the immunostaining pattern with NF200 and NK1r antibodies) before the CTb immunoreactivity was examined. For the rats that had received CVLM injections, sections from both ipsilateral and contralateral sides were analysed in this way (between three and nine sections per side per animal), and the depths of the cell bodies of all identified NF200-positive/NK1r-negative cells that had dorsal dendrites entering lamina II were measured. In order to test the effectiveness of the injections into the CVLM, we also determined the proportion of the large NK1r-immunoreactive lamina III/IV neurons on the contralateral side that were CTb-labelled in these experiments. In those rats that received gracile injections, only the left side of the L4 segment (ipsilateral to the targeted gracile nucleus) was analysed (five–eight sections per animal). In these experiments the depths of the cell bodies of a sample of CTb-labelled neurons were determined by examining all cells of this type in one section from the central part of the dorsal horn in each animal. Sections through the contralateral side of the L2 segments from the rats that had received injections into the thalamus were examined to determine whether any of the NF200-positive/NK1r-negative neurons with dendrites that entered the superficial dorsal horn were labelled with Fluorogold.

## Results

### Appearance of immunostaining with NF200, NK1r and substance P antibodies

**Fig. 1:**
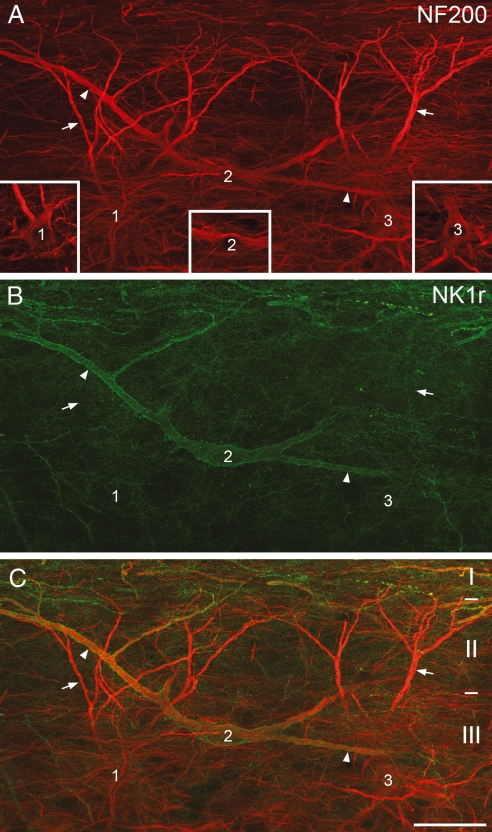
Immunostaining for neurofilament 200 (NF200) and the neurokinin 1 receptor (NK1r) in a parasagittal section through laminae I–III of the dorsal horn. Projections of confocal z-series show immunoreactivity for (A) NF200 and (B) NK1r, while a merged image is shown in (C). (A) NF200-immunoreactive structures, which include axons, dendrites and cell bodies, are present throughout the dorsal horn. Several strongly immunoreactive dendrites can be seen passing in a dorsoventral or oblique direction through lamina II. Analysis of the z-series revealed that these dendrites belong to three neurons, and the positions of their cell bodies are indicated with numbers (1–3). These cell bodies are not clearly seen in the projected images, but are shown in single optical sections or limited projections in the insets. (B and C) One of the three cells (2) is NK1 receptor-immunoreactive, and the immunostaining outlines the cell body and dendrites of this neuron (two of the dendrites are shown with arrowheads). The other two cells (1, 3) lack the NK1 receptor, and one dendrite belonging to each of these cells is indicated with arrows. Approximate positions of laminae are shown in (C). The main images are projections of 19 optical sections at 1 µm z-spacing. The insets were obtained from one, two or three optical sections, respectively. Scale bar: 50 µm.

NF200 immunoreactivity was present throughout both the white and grey matter of the spinal cord. Within the latter, staining was present in all laminae, but was much less intense in lamina II. Most NF200-positive profiles could readily be identified as axons or dendrites, based on their calibre and branching pattern. Dendrites showed a characteristic tapering appearance and branched frequently, with daughter branches being thinner than the parent branch. NF200 immunoreactivity was not seen in dendritic spines. NF200 immunostaining was also observed in cell bodies, but was weaker in these, presumably due to the more dispersed arrangement of neurofilaments. Although lamina II generally showed a low level of staining, it was frequently crossed by dendrites with strong NF200 immunoreactivity, which often branched extensively within it (Fig. 1A). These dendrites could usually be followed proximally to a cell body that was located ventral to lamina II. Because there was a much higher level of staining in deeper laminae, in many cases these cell bodies could only be identified with an oil-immersion lens. NF200-immunoreactive cell bodies were very rare in lamina II.

The pattern of NK1r immunostaining in laminae I–IV was the same as that seen in previous studies (Bleazard *et al*., 1994; Nakaya *et al*., 1994; Brown *et al*., 1995; Littlewood *et al*., 1995). Lamina I contained numerous immunoreactive cell bodies and dendrites, while lamina II contained fewer labelled structures, but was crossed by the dorsal dendrites of large NK1r-immunoreactive neurons that had cell bodies in lamina III or IV (Figs 1B and 3B).

Substance P immunoreactivity was present in axonal varicosities and intervaricose portions, and these were very numerous in the superficial dorsal horn, forming a dense plexus that occupied lamina I and the dorsal (outer) half of lamina II (IIo; Fig. 3C and G).

**Fig. 2:**
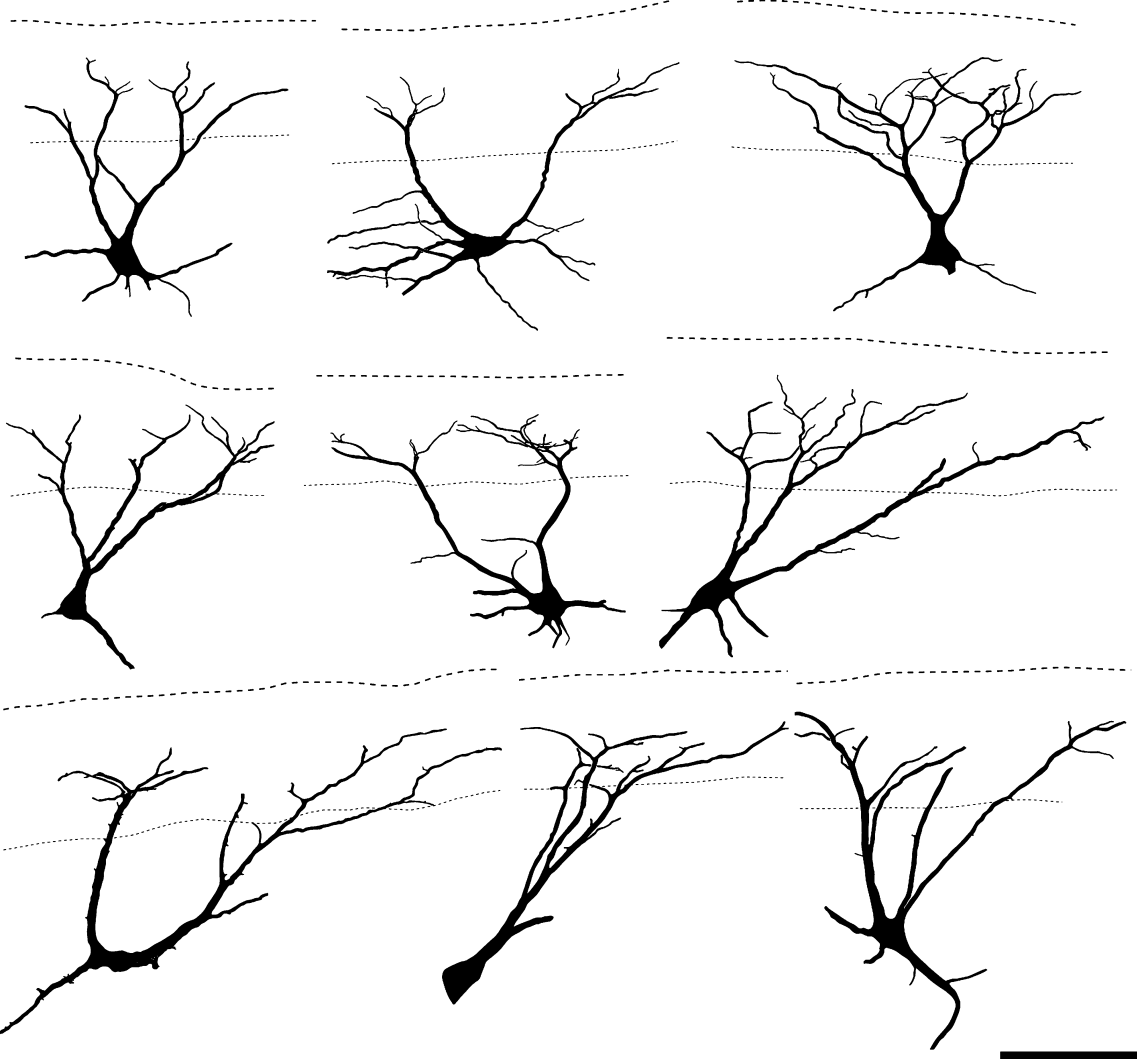
Drawings of six NF200-immunoreactive cells that lacked NK1 receptor-immunoreactivity (top and middle rows). For comparison, the bottom row shows three NK1 receptor-immunoreactive neurons. The drawing at the top left shows cell 1 in Fig. 1. In each case, the upper dashed line represents the dorsal limit of the grey matter, and the lower dashed line shows the ventral limit of the plexus of substance P-immunoreactive axons, which occupies laminae I and IIo. Scale bar: 100 µm.

When NF200 and NK1r immunoreactivities were examined together, it was apparent that all of the large lamina III/IV NK1r-immunoreactive neurons with long dorsal dendrites had NF200 immunoreactivity in their dendrites and cell bodies. Staining for NF200 was very strong in the dendritic trees of these neurons, and appeared to fill all of the dendrites that could be identified (Fig. 1, cell 2). However, it was also clear that many of the NF200-immunoreactive dendrites that passed dorsally through lamina II were not NK1r-immunoreactive (Fig. 1). Unless these dendrites left the section, they could invariably be seen to originate from cell bodies that were located deeper in the dorsal horn (Fig. 1, insets for cells 1 and 3). As with the NK1r-immunoreactive neurons, NF200 immunostaining in these NK1r-negative cells was very strong in proximal dendrites, could be followed into fine distal branches and appeared to occupy their full thickness. The depth of the cell body was measured for 27 NF200-positive/NK1r-negative cells that were examined in the three unoperated rats and for 112 such cells in the three rats that received CVLM injections. The mean depth below the white matter was 185 µm (range 100–307, SD 44). Although it is difficult to distinguish the lamina III/IV border in parasagittal sections, these depths correspond to lamina III and the dorsal part of lamina IV. The morphology of six representative cells of this type is shown in the upper and middle rows of Fig. 2, while three NK1r-immunoreactive lamina III neurons are illustrated in the lower row of Fig. 2. As can be seen by comparing these drawings (as well as those of NK1r-immunoreactive cells in figs 2 and 3 of Naim *et al*., 1997), the NF200-positive/NK1r-negative cells had a very similar appearance to the lamina III/IV NK1r-immunoreactive neurons that have been described previously (Bleazard *et al*., 1994; Nakaya *et al*., 1994; Brown *et al*., 1995; Littlewood *et al*., 1995; Naim *et al*., 1997). One or more dorsal dendrites passed up through laminae III and lamina II, before giving rise to numerous branches that occupied lamina IIo, and could sometimes be followed into lamina I (Fig. 2). Other primary dendrites could be seen leaving the cell body in ventral, rostral or caudal directions, but these were more difficult to follow due to the high density of NF200-immunoreactive profiles in laminae III and IV.

### Contacts between substance P axons and NF200-positive/NK1r-negative cells

**Fig. 3:**
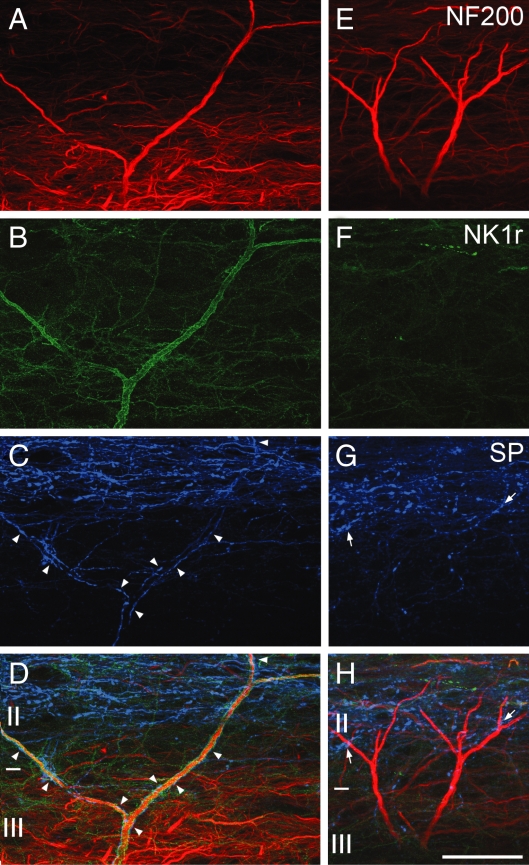
Contacts between substance P (SP)-immunoreactive varicosities and neurofilament 200 (NF200)-positive dendrites in laminae II and III. Two fields are shown, and these illustrate dendrites belonging to a neurokinin 1 receptor (NK1r)-positive/NF200-positive cell (A–D) and dendrites of a NK1r-negative/NF200-positive cell (E–H). In each case, NF200-immunoreactivity is shown in red (A, E), NK1r-immunoreactivity in green (B, F) and substance P-immunoreactivity in blue (C, G). (D) and (H) are merged images. (A–D) The dendrites of this NK1r-positive cell are surrounded by substance P-immunoreactive varicosities, which form numerous contacts in both laminae II and III (some of these are indicated with arrowheads). (E–H) In contrast, the dendrites of the NK1r-negative cell receive few contacts in the region ventral to the substance P plexus, although some contacts (two of which are indicated with arrows) are present on dendrites within the plexus. Approximate positions of laminae are shown in (D) and (H). The images are made from projections of 11 (A–D) or 15 (E–H) optical sections at 1 µm z-separation. Scale bar: 50 µm.

All of the cells that were analysed (15 cells, five per rat) received contacts from substance P-immunoreactive axonal varicosities, and these contacts were more numerous on those regions of the dendritic tree that lay within the substance P plexus (i.e. in laminae IIo or I) than on dendrites below the plexus (Fig. 3E–H). The mean density of contacts per 100 µm of dendrite was 10.42 ± 5.9 (SD) for superficial dendrites and 2.88 ± 2.38 for deep dendrites. We have previously analysed the density of contacts between substance P-immunoreactive varicosities and the dendrites of NK1r-immunoreactive lamina III/IV neurons, and found that these were 18.91 ± 4.04 per 100 µm for the superficial dendrites (within the substance P plexus), and 10.10 ± 4.04 for deep dendrites, with virtually all contacts being present on dendritic shafts of these neurons (Naim *et al*., 1997). The values obtained in the present study are significantly lower than those obtained for both superficial and deep dendrites of the NK1r-immunoreactive lamina III/IV cells (Naim *et al*., 1997; unpaired *t*-test, *P* < 0.001 for both superficial and deep dendrites).

A characteristic feature of the input to the lamina III/IV NK1r-immunoreactive neurons is that parts of the dendritic trees of these cells are surrounded by substance P-immunoreactive axons, the varicosities of which form numerous contacts on them (Naim *et al*., 1997). This is seen on both proximal and distal dendrites, but is more striking in lamina IIi and III, as the density of substance P-containing axons is relatively low in this region (Fig. 3A–D). This arrangement was never observed on the dendrites of the NF200-positive/NK1r-negative lamina III/IV cells (Fig. 3E–H).

### Retrograde labelling studies

**Fig. 4:**
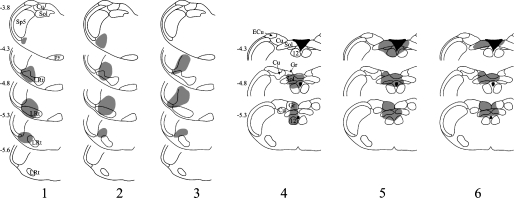
Diagrams to show the spread of CTb (shaded areas) following injections targeted on the CVLM (Experiments 1–3) or the gracile nucleus (Experiments 4–6). Each vertical column shows a series of drawings at different rostrocaudal levels of the brainstem from a single experiment. Numbers at the top left of each drawing in columns 1 and 4 give the approximate position of the section posterior to the ear-bar. Drawings are based on those of Paxinos & Watson (1997). 12, hypoglossal nucleus; Cu, cuneate nucleus; ECu, external cuneate nucleus; Gr, gracile nucleus; LRt, lateral reticular nucleus; py, pyramid; Sol, nucleus tractus solitarius; Sp5, spinal trigeminal nucleus.

**Fig. 5:**
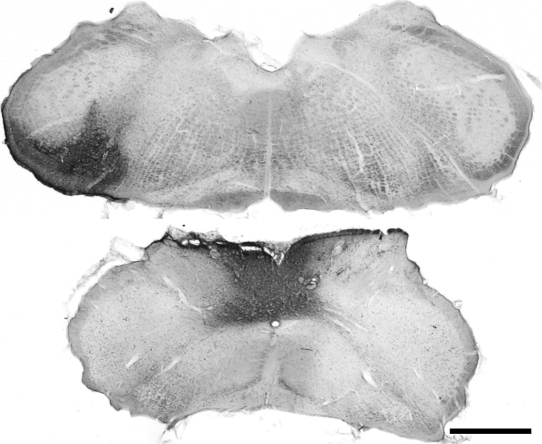
Examples of injection sites in the medulla. The upper image shows a section through an injection into the CVLM (in Experiment 1) and the lower image is from an injection into the gracile nucleus (Experiment 6). In each case, the left side of the brainstem is on the left. Scale bar: 1 mm.

**Fig. 6:**
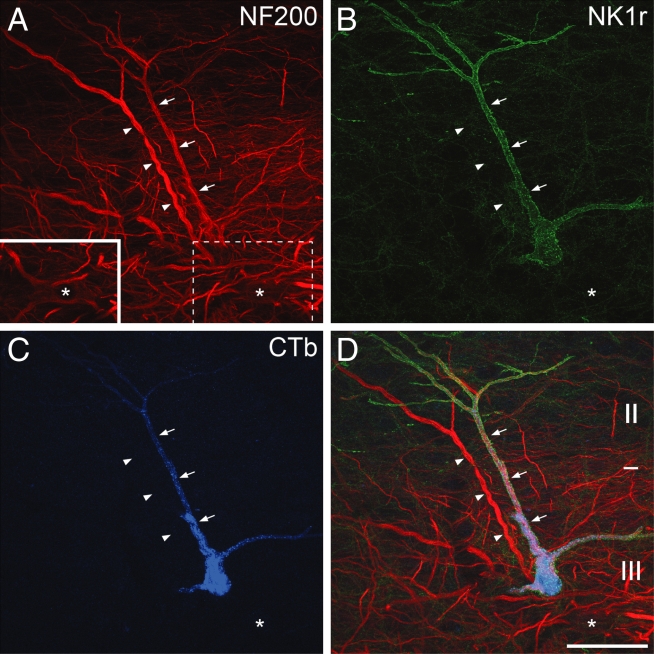
Immunostaining for (A) neurofilament 200 (NF200; red), (B) neurokinin 1 receptor (NK1r; green) and (C) cholera toxin B subunit (CTb; blue), together with a merged image (D) from the right (contralateral) side of the L4 segment following injection of CTb into the CVLM. The section is from the experiment illustrated in column 1 of Fig. 4. The upper half of the field corresponds to lamina II and the lower half to lamina III. (A) The NF200-positive dorsal dendrites of two different cells can be seen passing dorsally (one indicated with arrowheads and the other with arrows). One of these dendrites (arrows) is NK1r-immunoreactive (B) and contains CTb (C). The dendrites of this neuron can be seen to originate from a cell body in lamina III that is readily visible because of its NK1r- and CTb-immunolabelling (B**-**D). The other dorsal dendrite (arrowheads) is not NK1r-immunoreactive (B) and does not contain CTb (C). The cell body of this neuron is obscured in the main image, but can be seen in the inset in (A), which shows a projected image obtained from a more limited z-series. The location of the soma is shown by the asterisk, and the dashed line indicates the region shown in the inset. The soma of this neuron is also negative for NK1r and CTb (B–D). Approximate positions of laminae are shown in (D). Main images: projection of 19 optical sections at 1 µm z-separation; inset: projection of five optical sections at 1 µm z-separation. Scale bar: 50 µm.

Drawings of the spread of tracer within the brainstem after injection of CTb into the CVLM are shown in Fig. 4 (Experiments 1–3), and an example of an injection site is illustrated in Fig. 5. In each case the injection site occupied part of the region that lay medial to the spinal trigeminal nucleus and dorsal to the lateral reticular nucleus, with variable spread into these structures. In all three rats that had received CVLM injections, numerous retrogradely labelled neurons (identified by the presence of perikaryal CTb immunoreactivity) were present in lamina I, while CTb-labelled cells were also scattered through the deep dorsal horn (III–VI) and ventral horn. These cells were particularly numerous on the side contralateral to the injection. In all three cases, the majority of the large NF200-positive/NK1r-immunoreactive cells in laminae III and IV on the contralateral side of the L4 segment were CTb labelled (Table 1, Fig. 6). However, on the contralateral side none of the 55 NF200-positive/NK1r-negative cells with somata in laminae III or IV and dendrites that passed through lamina II was CTb labelled (Fig. 6), while only one out of the 57 cells of this type examined on the ipsilateral side was retrogradely labelled (Table 1).

**Table 1:** The proportions of NF200-immunoreactive lamina III/IV neurons that were CTb-labelled in the three CVLM injection experiments

Experiment number*	NK1r-negative, contralateral	NK1r-negative, ipsilateral	NK1r-positive, contralateral
1	0/22	0/19	16/20
2	0/11	0/17	20/20
3	0/22	1/21	15/25

* Experiment numbers refer to those in Fig. 4. NK1r, neurokinin 1 receptor.

Drawings of the spread of tracer for the three experiments in which CTb injections were targeted on the left gracile nucleus are illustrated in Fig. 4 (Experiments 4–6, and an example of an injection site is shown in Fig. 5). In all cases, there was extensive spread of tracer through the gracile nucleus at a level corresponding to 5.3 mm behind the interaural plane, although the nucleus was not filled at this level in one of the experiments (Experiment 4). There was a variable degree of spread of tracer into other structures on the ipsilateral side, such as the nucleus tractus solitarius and the hypoglossal nucleus, and across the midline (involving the contralateral nucleus tractus solitarius in Experiments 5 and 6). In each experiment, numerous retrogradely labelled cells were present in laminae III–V of the left dorsal horn in the L4 segment in all sections examined. A single section from each experiment was used to determine the depths of the retrogradely labelled cells, and in these sections the total numbers of cells counted were 46, 39 and 51 for Experiments 4–6, respectively. The depths of cells below the white matter ranged from 215 to 553 µm, with a mean value of 342 (± 63, SD, data pooled from three animals). These values are significantly different from those obtained for the NF200-positive/NK1r-negative cells (*P <* 0.001, two-sample *t*-test). Fifty-six NF200-positive/NK1r-negative lamina III/IV neurons with dendrites that entered the superficial dorsal horn were identified in sections from these rats (13–26 per experiment). None of these cells was retrogradely labelled with CTb.

**Fig. 7:**
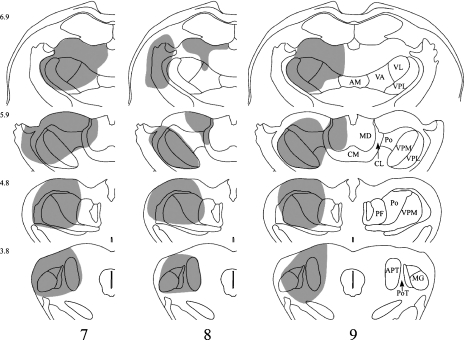
Diagrams to show the spread of Fluorogold (shaded areas) following injections into the thalamus (Experiments 7–9). Each vertical column shows a series of drawings at different rostrocaudal levels of the brain from a single experiment. The numbers at the top left of each drawing give the approximate position of the section anterior to the ear-bar. The drawings are based on those of Paxinos & Watson (1997). AM, anteromedial thalamic nucleus; APT, anterior pretectal nucleus; CL, centrolateral thalamic nucleus; CM, central median thalamic nucleus; MD, mediodorsal thalamic nucleus; MG, medial geniculate nucleus; PF, parafascicular thalamic nucleus; Po, posterior thalamic nuclear group; PoT, posterior thalamic nuclear group, triangular part; VA, ventral anterior thalamic nucleus; VL, ventrolateral thalamic nucleus; VPL, ventral posterolateral thalamic nucleus; VPM, ventral posteromedial thalamic nucleus.

**Fig. 8:**
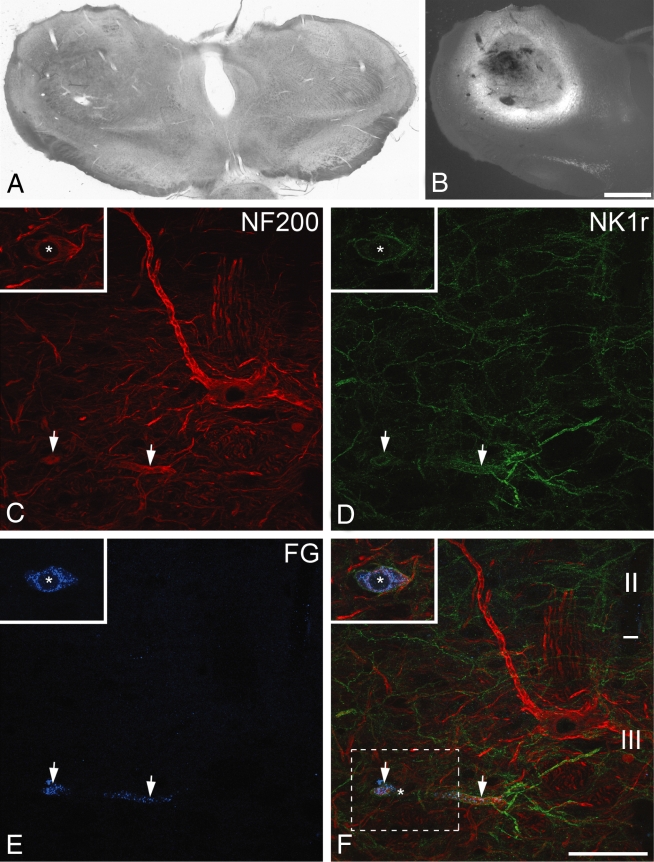
Examples of an injection site in the thalamus and lack of retrograde labelling of a neurokinin 1 receptor (NK1r)-negative/neurofilament 200 (NF200)-positive lamina III cell. (A) A coronal section through the thalamus at approximately 4.5 mm anterior to the ear-bar. (B) A fluorescence micrograph of the left-hand side of the section seen in (A) to show the spread of Fluorogold (FG). This section is taken from Experiment 8. (C–F) Immunostaining for NF200 (red), NK1r (green) and FG (blue), together with a merged image from the right (contralateral) side of the L2 segment after injection of the tracer into the thalamus (Experiment 7). The main images show a region that contains a large NK1r-negative/NF200-positive cell, which is not labelled with FG. Proximal dendrites of a retrogradely labelled NK1r-positive neuron are also visible (arrows). The cell body of this neuron (asterisk) was located deeper in the Vibratome section, and scans through this are shown in the insets. The dashed line in (F) shows the area represented in these insets. Approximate positions of laminae are indicated in (F). (C–F) The main images are projections of seven optical sections at 1 µm z-separation, while those in the inset are projections of five optical sections at 1 µm z-separation. Scale bars: 1 mm (A and B); 50 µm (C–F).

Injections of Fluorogold into the thalamus (Figs 7 and 8A and B) completely filled the triangular part of the posterior group of nuclei (PoT) in all three cases, and occupied all (Experiments 7 and 9) or most (Experiment 8) of the main part of the posterior group (Po). The injection site covered virtually all of the ventral posterolateral nucleus at 5.9 mm anterior to the ear-bar in all three experiments, although it did not extend into this nucleus further rostrally in one case (Experiment 8). Injections included part of the mediodorsal nucleus in two cases (Experiments 7 and 9). Fluorogold-positive neurons were seen in lamina I and deeper laminae (III–VI) in the contralateral dorsal horn. The number of retrogradely labelled NK1r-immunoreactive neurons in laminae III–IV ranged from four to 10 in the three animals. Fifty-five NF200-positive/NK1r-negative lamina III/IV neurons with dendrites that entered the superficial dorsal horn were identified in sections from these rats (15–22 per experiment), and none of these cells was retrogradely labelled with Fluorogold **(**Fig. 8C–E**)**.

## Discussion

The results of the present study clearly demonstrate that there are two different populations of large dorsal horn neurons that have cell bodies in lamina III or IV and long dorsal dendrites that pass into the superficial laminae: (1) those that express the NK1r, which have been identified in several previous studies (Bleazard *et al*., 1994; Nakaya *et al*., 1994; Brown *et al*., 1995; Littlewood *et al*., 1995); and (2) a population that has not been recognized before and consists of neurons that lack the NK1r. Neurons in this second population have certain features in common with the NK1r-expressing cells: (1) they both show high levels of NF200 expression in their dendrites; (2) they have dendritic trees that are morphologically indistinguishable; and (3) their cell bodies are located at similar depths below the white matter (mean depth of 185 µm for the NK1r-negative cells, compared with a mean depth of 209 µm for the NK1r-expressing neurons; Polgar *et al*., 2007)*.* However, there are also major differences between the two populations, not only in terms of NK1r expression, but also concerning their position in the neuronal circuitry of the dorsal horn. Firstly, the NK1r-negative cells received significantly fewer contacts from substance P-containing axons, and never had the close associations with individual substance P axons that are a characteristic feature of the NK1r-immunoreactive cells (Naim *et al*., 1997)*.* Secondly, none of these cells was retrogradely labelled following injection of CTb into the contralateral CVLM, whereas the great majority of the large NK1r-expressing lamina III/IV neurons can be labelled from this region (Todd *et al*., 2000; present study)*.*

### Retrograde labelling studies

We looked for evidence that the lamina III/IV NF200-positive/NK1r-negative cells were retrogradely labelled from the contralateral CVLM, because we have previously shown that over 90% of the large NK1r-immunoreactive neurons in these laminae could be labelled from injections into this region (Todd *et al*., 2000)*.* In two of the CVLM experiments in the present study we found that, as expected, the great majority of these NK1r-immunoreactive cells were CTb-positive (Table 1, Experiments 1 and 2). In the remaining experiment, only 15 out of 25 (60%) of the NK1r-immunoreactive cells were retrogradely labelled, and this may be because the injection site was slightly more dorsal in this case. However, although this injection may not have been optimal, the results from these three experiments clearly indicate that the lamina III/IV NF200-positive/NK1r-negative neurons with long dorsal dendrites differ from the NK1r-positive cells, in that they are not retrogradely labelled from the contralateral CVLM.

During the course of a study in which we investigated projections to the periaqueductal grey matter, the lateral parabrachial area and the CVLM (Todd *et al*., 2000)*,* we observed very few retrogradely labelled NK1r-negative neurons in laminae III and IV on the contralateral side after injection into any of these sites. However, we did find a significant number of labelled cells in laminae III and IV that lacked the receptor on the ipsilateral side after the CVLM injections (Fig. 4 of Todd *et al*., 2000)*.* We therefore examined sections from the ipsilateral side in each of the CVLM experiments in the present study, but found that only one of the NF200-positive/NK1r-negative lamina III/IV cells was retrogradely labelled in one experiment. Although retrogradely labelled NK1r-negative neurons were seen on the ipsilateral side in these experiments, they did not have dendrites that could be followed into the superficial dorsal horn.

Because there is a significant projection from the deep dorsal horn to the thalamus, we also tested whether the lamina III/IV NF200-positive/NK1r-negative cells belonged to the spinothalamic tract, by injecting Fluorogold into its major target areas (Gauriau & Bernard, 2004; Willis & Coggeshall, 2004)*.* However, although retrogradely labelled neurons were seen in laminae III–VI, none of them belonged to this population.

Another potential projection target for this population of cells is the gracile nucleus. In the rat lumbar spinal cord, PSDC cells (which project to the ipsilateral gracile nucleus) are found in large numbers in an area of the dorsal horn that corresponds to laminae III–V (Giesler *et al*., 1984), and it has been shown that these cells do not express the NK1r (Polgár *et al*., 1999; Palecek *et al*., 2003)*.* In addition, studies of intracellularly labelled PSDC cells in the cat have demonstrated that some of those that have their cell bodies in lamina III have dendrites that can extend dorsally as far as lamina I (Brown & Fyffe, 1981)*.* However, we found that although numerous retrogradely labelled neurons were present in the ipsilateral dorsal horn after injections that included the gracile nucleus, none of these belonged to the population of NF200-positive/NK1r-negative cells with long dorsal dendrites. In fact, although we found some labelled cells in lamina III, the majority of them appeared to be located ventral to the main region in which this population of neurons was found. This is consistent with the findings of Giesler *et al*. (1984)*,* who showed that most PSDC cells are located well below the ventral limit of the substantia gelatinosa. Because two of the gracile injections resulted in partial filling of the nucleus tractus solitarius on both sides, it is very unlikely that these cells project to this nucleus.

Our results therefore indicate that cells of this type do not project to the ipsilateral gracile nucleus, the contralateral thalamus or to the CVLM or nucleus tractus solitarius on either side. Our previous findings suggest that they are also unlikely to project to the periaqueductal grey matter or lateral parabrachial area (Todd *et al*., 2000)*.* Because these sites represent the major projection targets of dorsal horn neurons, it seems likely that these cells do not have supraspinal projections. Because they are relatively large neurons, it is likely that they have long axons that could be involved in propriospinal connections.

### Potential primary afferent inputs

Neurons with cell bodies in lamina III or IV and dorsal dendrites that enter the superficial dorsal horn provide a route through which information transmitted by nociceptive primary afferents, most of which terminate in laminae I and II, can be conveyed to deeper laminae, and elsewhere in the CNS*.*Szentágothai (1964) proposed that cells of this type represented the output of the substantia gelatinosa, which at that time was thought to be a closed system.

The lamina III/IV NK1r-immunoreactive neurons clearly perform this function, as they receive a strong monosynaptic input from substance P-containing primary afferents (Naim *et al*., 1997)*.* In fact, this input is not restricted to laminae I and IIo, where most peptidergic afferents terminate, but extends ventrally to reach their proximal dendrites in laminae IIi and III. In the present study, we found that all of the NF200-positive/NK1r-negative neurons with long dorsal dendrites received contacts from substance P-immunoreactive axons, and it is likely that many of these axons were of primary afferent origin. However, we have previously observed contacts between substance P-containing primary afferents and interneurons that express the µ opioid receptor MOR1, which were apparently not associated with synapses (Spike *et al*., 2002)*.* Electron microscopy would therefore be required to determine whether the substance P-containing axons form synapses with the lamina III/IV NF200-positive/NK1r-negative cells.

Another major group of unmyelinated primary afferents consists of C fibres that appear to lack neuropeptides (Todd & Koerber, 2005)*.* These afferents possess cell surface α-d-galactose residues that bind *Bandeirea simplicifolia* isolectin B4 (BSI-B4; Silverman & Kruger, 1990; Wang *et al*., 1994), and can be identified by their ability to bind the lectin, together with the lack of calcitonin gene-related peptide (CGRP) immunoreactivity (Ju *et al*., 1987; Sakamoto *et al*., 1999)*.* Non-peptidergic afferents are thought to function as nociceptors (Guo *et al*., 1999; Michael & Priestley, 1999; Stucky & Lewin, 1999) and they are known to give rise to the central axons of type I synaptic glomeruli, which are found within lamina II (Ribeiro-da-Silva & Coimbra, 1982, Rideiro-da-Siva *et al*., 1986; Gerke & Plenderleith, 2004)*.* Interestingly, the NK1r-immunoreactive lamina III/IV neurons with long dorsal dendrites do not appear to receive significant input from non-peptidergic C fibres, as their dendrites were found to have very few contacts from varicosities that bound BSI-B4 but lacked CGRP (Sakamoto *et al*., 1999)*.*Todd (1989) provided evidence that the dorsal dendrites of lamina III/IV cells with a similar morphology to those examined in the present study received numerous primary afferent synapses in lamina II, and that at least some of these were from the central axons of synaptic glomeruli, although it is not known whether the sample of cells examined in that study included any that lacked the NK1r. It is possible that the NF200-positive/NK1r-negative lamina III/IV cells receive monosynaptic input from non-peptidergic C fibres in lamina II, and this may explain the finding of glomerular synapses on the dendrites of some of the cells studied by Todd (1989)*.* Because the NF200 antibody labels dendritic shafts, but does not extend into spines, we are unable to determine whether these neurons possess dendritic spines. A significant part of the primary afferent input to lamina III/IV neurons is located on dendritic spines (Todd, 1989), and these form the major postsynaptic component in synaptic glomeruli (Ribeiro-da-Silva & Coimbra, 1982). It would therefore not be possible to assess the extent of the input from non-peptidergic C fibres to NF200-positive/NK1r-negative cells with confocal microscopy.

## Abbreviations

BSI-B4: *Bandeirea simplicifolia* isolectin B4
CGRP: calcitonin gene-related peptide
CTb: cholera toxin B subunit
CVLM: caudal ventrolateral medulla
NF200: neurofilament 200
NK1r: neurokinin 1 receptor
PSDC: postsynaptic dorsal column.

## References

[b1] Bleazard L, Hill RG, Morris R (1994). The correlation between the distribution of the NK1 receptor and the actions of tachykinin agonists in the dorsal horn of the rat indicates that substance P does not have a functional role on substantia gelatinosa (lamina II) neurons. J. Neurosci..

[b2] Brown AG (1981). Organization in the Spinal Cord: the Anatomy and Physiology of Identified Neurones.

[b3] Brown AG, Fyffe RE (1981). Form and function of dorsal horn neurones with axons ascending the dorsal columns in cat. J. Physiol..

[b4] Brown JL, Liu H, Maggio JE, Vigna SR, Mantyh PW, Basbaum AI (1995). Morphological characterization of substance P receptor-immunoreactive neurons in the rat spinal cord and trigeminal nucleus caudalis. J. Comp.Neurol..

[b5] Cuello AC, Galfre G, Milstein C (1979). Detection of substance P in the central nervous system by a monoclonal antibody. Proc.Natl Acad.Sci.USA.

[b6] Gauriau C, Bernard JF (2004). A comparative reappraisal of projections from the superficial laminae of the dorsal horn in the rat: the forebrain. J. Comp.Neurol..

[b7] Gerke MB, Plenderleith MB (2004). Ultrastructural analysis of the central terminals of primary sensory neurones labelled by transganglionic transport of bandeiraea simplicifolia I-isolectin B4. Neuroscience.

[b8] Giesler GJ, Nahin RL, Madsen AM (1984). Postsynaptic dorsal column pathway of the rat. I. Anatomical studies. J. Neurophysiol..

[b9] Guo A, Vulchanova L, Wang J, Li X, Elde R (1999). Immunocytochemical localization of the vanilloid receptor 1 (VR1): relationship to neuropeptides, the P2X3 purinoceptor and IB4 binding sites. Eur.J.Neurosci..

[b10] Hokfelt T, Kellerth JO, Nilsson G, Pernow B (1975). Substance P: localization in the central nervous system and in some primary sensory neurons. Science.

[b11] Ju G, Hokfelt T, Brodin E, Fahrenkrug J, Fischer JA, Frey P, Elde RP, Brown JC (1987). Primary sensory neurons of the rat showing calcitonin gene-related peptide immunoreactivity and their relation to substance P-, somatostatin-, galanin-, vasoactive intestinal polypeptide- and cholecystokinin-immunoreactive ganglion cells. Cell Tissue Res..

[b12] Lawson SN, Crepps BA, Perl ER (1997). Relationship of substance P to afferent characteristics of dorsal root ganglion neurones in guinea-pig. J. Physiol..

[b13] Lima D, Mendes-Ribeiro JA, Coimbra A (1991). The spino-latero-reticular system of the rat: projections from the superficial dorsal horn and structural characterization of marginal neurons involved. Neuroscience.

[b14] Littlewood NK, Todd AJ, Spike RC, Watt C, Shehab SA (1995). The types of neuron in spinal dorsal horn which possess neurokinin-1 receptors. Neuroscience.

[b15] Llewellyn-Smith IJ, Minson JB (1992). Complete penetration of antibodies into vibratome sections after glutaraldehyde fixation and ethanol treatment: light and electron microscopy for neuropeptides. J. Histochem.Cytochem..

[b16] Mantyh PW, DeMaster E, Malhotra A, Ghilardi JR, Rogers SD, Mantyh CR, Liu H, Basbaum AI, Vigna SR, Maggio JE, Simone DA (1995). Receptor endocytosis and dendrite reshaping in spinal neurons after somatosensory stimulation. Science.

[b17] Marshall GE, Shehab SAS, Spike RC, Todd AJ (1996). Neurokinin-1 receptors on lumbar spinothalamic neurons in the rat. Neuroscience.

[b18] McLeod AL, Krause JE, Ribeiro-Da-Silva A (2000). Immunocytochemical localization of neurokinin B in the rat spinal dorsal horn and its association with substance P and GABA: an electron microscopic study. J. Comp.Neurol..

[b19] Michael GJ, Priestley JV (1999). Differential expression of the mRNA for the vanilloid receptor subtype 1 in cells of the adult rat dorsal root and nodose ganglia and its downregulation by axotomy. J. Neurosci..

[b20] Molander C, Xu Q, Grant G (1984). The cytoarchitectonic organization of the spinal cord in the rat. I. The lower thoracic and lumbosacral cord. J. Comp.Neurol..

[b21] Naim M, Spike RC, Watt C, Shehab SA, Todd AJ (1997). Cells in laminae III and IV of the rat spinal cord that possess the neurokinin-1 receptor and have dorsally directed dendrites receive a major synaptic input from tachykinin-containing primary afferents. J. Neurosci..

[b22] Nakaya Y, Kaneko T, Shigemoto R, Nakanishi S, Mizuno N (1994). Immunohistochemical localization of substance P receptor in the central nervous system of the adult rat. J. Comp.Neurol..

[b23] Palecek J, Paleckova V, Willis WD (2003). Postsynaptic dorsal column neurons express NK1 receptors following colon inflammation. Neuroscience.

[b24] Paxinos G, Watson C (1997). The Rat Brain in Stereotaxic Coordinates.

[b25] Polgar E, Campbell AD, MacIntyre LM, Watanabe M, Todd AJ (2007). Phosphorylation of ERK in neurokinin 1 receptor-expressing neurons in laminae III and IV of the rat spinal dorsal horn following noxious stimulation. Mol.Pain.

[b26] Polgár E, Furuta T, Kaneko T, Todd AJ (2006). Characterization of neurons that express preprotachykinin B in the dorsal horn of the rat spinal cord. Neuroscience.

[b27] Polgár E, Shehab SAS, Watt C, Todd AJ (1999). GABAergic neurons that contain neuropeptide Y selectively target cells with the neurokinin 1 receptor in laminae III and IV of the rat spinal cord. J. Neurosci..

[b28] Ramon y Cajal S (1909). Histologie Du Système Nerveux de L'homme et Des Vertébrés, I.

[b29] Réthelyi M, Szentágothai J (1969). The large synaptic complexes of the substantia gelatinosa. Exp.Brain Res..

[b30] Rexed B (1952). The cytoarchitectonic organization of the spinal cord in the cat. J. Comp.Neurol..

[b31] Ribeiro-da-Silva A, Castro-Lopes JM, Coimbra A (1986). Distribution of glomeruli with fluoride-resistant acid phosphatase (FRAP)-containing terminals in the substantia gelatinosa of the rat. Brain Res..

[b32] Ribeiro-da-Silva A, Coimbra A (1982). Two types of synaptic glomeruli and their distribution in laminae I–III of the rat spinal cord. J. Comp.Neurol..

[b33] Sakamoto H, Spike RC, Todd AJ (1999). Neurons in laminae III and IV of the rat spinal cord with the neurokinin-1 receptor receive few contacts from unmyelinated primary afferents which do not contain substance P. Neuroscience.

[b34] Scheibel ME, Scheibel AB (1966). Terminal axonal patterns in cat spinal cord. I. The lateral corticospinal tract. Brain Res..

[b35] Schoenen J (1982). The dendritic organization of the human spinal cord: the dorsal horn. Neuroscience.

[b36] Silverman JD, Kruger L (1990). Selective neuronal glycoconjugate expression in sensory and autonomic ganglia: relation of lectin reactivity to peptide and enzyme markers. J. Neurocytol..

[b37] Spike RC, Puskar Z, Sakamoto H, Stewart W, Watt C, Todd AJ (2002). MOR-1-immunoreactive neurons in the dorsal horn of the rat spinal cord: evidence for nonsynaptic innervation by substance P-containing primary afferents and for selective activation by noxious thermal stimuli. Eur.J.Neurosci..

[b38] Stucky CL, Lewin GR (1999). Isolectin B(4)-positive and -negative nociceptors are functionally distinct. J. Neurosci..

[b39] Szentágothai J (1964). Neuronal and synaptic arrangement in the substantia gelatinosa Rolandi. J. Comp.Neurol..

[b40] Todd AJ (1989). Cells in laminae III and IV of rat spinal dorsal horn receive monosynaptic primary afferent input in lamina II. J. Comp.Neurol..

[b41] Todd AJ, Koerber HR, McMahon S, Koltzenburg M (2005). Neuroanatomical substrates of spinal nociception. Wall and Melzack's Textbook of Pain.

[b42] Todd AJ, McGill MM, Shehab SA (2000). Neurokinin 1 receptor expression by neurons in laminae I, III and IV of the rat spinal dorsal horn that project to the brainstem. Eur.J.Neurosci..

[b43] Wang H, Rivero-Melian C, Robertson B, Grant G (1994). Transganglionic transport and binding of the isolectin B4 from Griffonia simplicifolia I in rat primary sensory neurons. Neuroscience.

[b44] Willis WD, Coggeshall RE (2004). Sensory Mechanisms of the Spinal Cord, *Vol. 2: Ascending Sensory Tracts and Their Descending Control*.

